# False Impressions

**Published:** 1889-03-02

**Authors:** Caroline Fothergill

**Affiliations:** Author of "An Enthusiast," "A Voice in the Wilderness," etc.


### FALSE IMPRESSIONS.

By Caroline Fothergill, Author of "An Enthusiast," "A Voice in the Wilderness," etc.

(*Continued from p.* 336.)

### Chapter XXI.—Switzerland.

Long years after everyone else had forgotton all about it, Edith remembered that Swiss journey. It lasted a month, and during the whole of that month she was perfectly happy. The whole time was like a dream, each day more perfect than the last. She was weak when she left home, and the journey, taken in easy stages though it was, taxed her strength severely, so that she needed a few days' rest before she could begin to enjoy herself. When she could walk and drive, she found that Switzerland surpassed her wildest expectations, if "wild" be not too strong an expression to apply to any of Edith's feelings. The beauty, grandeur, and wildness of the scenery at first overpowered and depressed, then charmed and fascinated her, beyond anything she had ever imagined, and she presently grew to love it with all her heart. The busy hotel life, the throng of Americans and the twang of their speech also amused and interested her. She would have felt shy and uncomfortable if George had not been always at her side to shield and protect her from these dreaded strangers. He put himself entirely at her service, and was constantly suggesting fresh schemes to give her pleasure. Mrs. Lester, too, was unusually kind, and left them a great deal to themselves, and with the belief she had given to Edith before she left home it was the kindest thing she could do.

Edith had accepted what her aunt said without any question; it chimed in too well with her own desires to be rejected. It is true that from time to time the recollection of Miss Stanford came to disturb her. The scene at the museum, Mr. Murray's attentions to her at his own house, and the remarks Harry Fairfax had made about them; the thought ef these things always made her uncomfortable, but with the ease of youth and ignorance she was always able to throw off such thoughts. Of course, she argued, Mr. Murray had to pay due attention to Miss Stanford, a lady of wealth and position: he corld not do otherwise. And no doubt he admired her—she was too beautiful not to be admired; but it was she, little Edith, who had his heart. And then she blushed and hid her face from her own consciousness. She was in a state of blissful anticipation, by no means impatient. She felt as though she would be content to let things go on for ever in this way. She was in no hurry for George to speak, she could scarcely credit the immense happiness which had fallen to her share, and her mind was constantly busy with plans of what she would do for her father and the boys when she should have been changed from a poor little girl to a wealthy lady of importance. Sometimes she thought of Harry Fairfax as of a being belonging to another world; but her thoughts of him had a lingering, half-sad affection, such as we may imagine would be felt by those who having attained heaven, look down upon those with whom they lived and loved upon earth.

Her manner all this time was very charming. It had gained in dignity and consciousness, but was still very girlish and yielding at times, and Mrs. Lester, watching her critically, could find no flaw in it, and she wondered within herself how the child managed it.

George enjoyed himself, too; he was very fond of little Edith, and liked, above all things, to give her pleasure. It gratified him as much in one way to see her face flush with shy delight at some kindness on his part, as it did in another, to see the colour mount up to Beatrice Stanford's cheek as she averted her face from him sometimes. She was so quiet and unexacting that he could take her for a walk or a drive, and enjoy her presence while his thoughts wandered very far from her He never spoke of Beatrice to Edith. He was naturally reserved, and although he had made such a pet and companion of her, she had no entrance to his inner life; neither did she often occupy his thoughts when he was absent from her.

So far, things had gone smoothly enough. Mrs. Lester, if she was not quite happy, dared say nothing. Things were not moving in a way altogether satisfactory to her. She had no fault to find with Edith, but her brother was a sore trial to her. She had brought these two away, and left them constantly together. On account of Edith, who often got tired in the evening, and could not bear the heat and noise of the public drawing-room, they had a private sitting room, and George and Edith spent nearly every evening there. But George, though kindness itself, was friendly, and no more. He was no more in love with Edith now than he had been when they left home. He did not even seem to notice Edith's affection for himself, and although Mrs. Lester had given him as broad a hint on the subject as she dared, he had taken no notice at all of it; he had not even seemed to know what she meant. She clenched her hands in disappointment and anger when she was alone. Surely her scheme was not going to fail; it could not, should not; she was capable of committing some desperate deed to secure her own ends, and, so far as she could tell, nothing of any consequence happened. The only thing of any significance never came to her knowledge.

George and Edith were alone in the sitting-room. Mrs. Lester for various reasons preferred the public drawing-room. They had been talking about a long drive which they had taken that day, and by degrees the light had faded, until it was quite dusk. They had ceased talking some time since, and George's thoughts had gone off to their favourite subject. Edith, watching his face, saw that he was thinking deeply about something, and his expression made her think it must be about something pleasant, and gave her the courage which she still sometimes needed to break in upon his reflections.

"What are you thinking about?" she asked softly.

He turned and looked at her as she lay in the dim light. He by no means felt inclined to open his heart to her, but some hint of the impending change might be given. He had thought about her in connexion with his future, and had supposed she must go with Frances. It would be a dull life, but he and Beatrice would often have her with them, and contrive treats and holidays for her. He was sure she would not like leaving home; a little preparation might not come amiss; so after looking at her for a minute in silence, he said—

"I was thinking about the future, Edith. Suppose I were to get married. What should you say to that?"

Her heart began to beat very fast, and her head swam. She believed that he was going to ask her to be his wife now, and although she was too agitated to understand the significance of it, as soon as she thought this, all her love vanished, and she only felt frightened out of her senses, far too frightened to speak.

"Your silence is not very encouraging," he said at last, as she did not speak. "What am I to understand by it? Is it that you dislike the idea so much?"

"Oh, no," she said quickly, and speaking scarcely above her breath.

He was a little surprised at her reply, but said, half-jestingly—

"No? That is very good of you. I was half afraid you would not like the idea, and I hesitated about mentioning it to you; but I see that I need not have done."

It was not very ardent wooing, but such as it was it so overpowered Edith that she again said nothing, being unable to speak. This question so looked forward to in advance, so dreaded, now she believed it had come, quite took away her breath.

"But however you feel about it," he went on; "and it is just like you to make no difficulty; I am afraid I must expect opposition from Frances. She will not like it at all."

"Oh, yes," said Edith, feeling obliged to speak at this point. "She is very kind; she says she will like it very much."

George looked at her in undisguised astonishment. He had not been prepared for this. His treasured secret was then no secret at all; his chances had been weighed by these two over their work, or their teacups. The idea was galling to him, and took away all desire for further confidence. He answered rather drily:

"I did not know I had shown my intentions so very plainly, or that my affairs had already been discussed. May I ask if it is long since my sister told you she would be pleased at my marriage?"

"It was the night before we left home," said Edith, feeling very much alarmed.

"I see. I had not expected such generosity from her."

Edith said nothing, but only lay there feeling miserably uncomfortable, and George got up and took one or two turns in the room. Then he stopped by the couch and said :

"Never mind, Edith, you must not be distressed ; it will all come right. But be good enough not to report any of our conversation to your aunt."

Edith dried her tears which had begun to flow, and said no more. This talk rather puzzled her. She did not know why Mr. Murray had not spoken more explicitly, but supposed he had good reasons for not doing so ; and on the whole she was glad. She liked things as they were now.

A week later they turned their steps homewards, travelling slowly, for no one, except George, was in haste to reach home, and he curbed his impatience with philosophy.

Mayfield seemed very dingy after Switzerland, but the intense heat was over, and, Edith being now quite strong again, took up all her old employments—her district visiting and calls on old Mrs. Jones. The old woman was delighted to see her again, and told her all that had happened since she had gone away. The chief events were the almost daily calls from Harry Fairfax, of whose kindness Mrs. Jones could not speak too highly. She believed there never had been such a young gentleman. He was like no curate she had ever known before, and though in her enthusiasm she did once speak of him as "that young Harry," on observing the rather shocked expression on Edith's face, she skilfully returned to her former flattering mode of speech. It appeared, too, that he was for ever talking about Miss Lester—the questions he had asked about her were beyond Mrs. Jones's power of enumeration.

The second time Edith went Harry was there, and insisted on walking home with her. He expatiated upon the improvement in Edith's appearance, and felt almost as grateful to Mr. Murray as she was herself for the evident good she had got from her journey. There was a little difference in her manner, too; it was calmer and more assured than formerly. She was very glad to see Harry again. She could not be blind to his sterling qualities and soundness of character, but now she looked upon him no more than a kind and valued friend.

They talked for some time, and then the conversation languished. Harry had not his usual flow of words—he was nervous and halting in his speech. Edith, little thinking what was coming, wondered what could be the matter with him, and was struck almost dumb with astonishment when she discovered that he was asking her to marry him.

"I know you don't love me as I love you," he was saying, "but I don't expect that now ; I would be patient and wait. But if you would only care for me a little—enough to trust me to make you happy, and to think of you first, all my life, I would be content.'

It was honest, manly pleading, and touched Edith deeply, even though she made no answer to it.

"I know I am not worthy of you," he went on, "and a curate has not much of a position to offer his wife ; but I will do my very best to deserve you, and I will work hard to get promotion. I promise you shan't be a curate's wife all your life. I have some influence, and I am sure Mr. Murray would do what he could for us."

The mention of George's name brought Edith back to herself.

"I am very sorry," she began, "It very kind of you to ask me to be your wife, and I am very much obliged to you. I like you very much too, but I really can't marry you. Please, don't ask me."

Harry would not take such an answer. It was a horrible disappointment to him ; he had almost counted upon being accepted, and he could not believe that all his hopes were to be crushed in this way. He besought Edith to think again: not to be in a hurry—he would not press her for an answer now. He would like her to go home and thinkabout it, and write to him when she had made up her mind.

It was all in vain. Edith was terribly distressed. She shed tears, and felt perfectly miserable, but she did not yield. She told him decidedly that she could not think of him as a husband and at last induced him to leave her and let her finish her walk home alone.

When she reached home, she sought her room at once, and for an hour lay on her bed, bathed in tears, and full of pity and sympathy for Harry. She did like him, only now she knew how much ; she felt at times as if she could have been happy as his wife, and then the thought of George came over her. He, too, had spoken to her of marriage, and she had not said him nay. She reproached herself with fickleness, and hardened her heart against Harry.

George was out that evening, so was spared the sight of her tear-stained face ; but Mrs. Lester saw it, and inquired as to its cause. Edith, never clever at evading questions, had told her story almost before she knew what she was saying, and it must be owned that Mrs. Lester felt a twinge of conscience when she heard. But she said nothing till Edith concluded with—

"I was right to refuse him, was I not, aunt ?"

"Certainly, if you did not care for him," said her aunt, with some constraint.

"I do like him very much ; but you know, aunt," she went on timidly, "there is someone else."

To this Mrs. Lester made no reply.

*(To be continued.)*

